# Supplementary material for: Characterization of the Bax Inhibitor-1 Family in Cauliflower and Functional Analysis of BobBIL4
Source: Int J Mol Sci. 2024 Sep 3;25(17):9562. doi: 10.3390/ijms25179562 (PMC11395134; doi:10.3390/ijms25179562)
Supplement: Supplementary file 1 [file ijms-25-09562-s001.zip › ijms-3137846-supplementary.pdf]

```
BobBIL4      ............MWN..QKY...DLES....AQTPLYPMMMESP.ELRWSFIRKVYSIISIQLLVTIAVA     48
BobLFG5.3    MEKPYGYASVSMSMSGVDRSAGKDIDLEMG..EATLYPGLSYGENQLRWGFIRKVYGILSAQLLLTTLIS     68
BobLFG5.1    MEKPYGYASVGMSS..VDRAAGKDIDLEMGGGEATLYPGLSYGENQLRWGFIRKVYGILSAQLLLTTLIS     68
BobLFG5.2    MEKPYGYASVGMSS..VDRAAGKDIDLEMGGGEATLYPGLSYGENQLRWGFIRKVYGILSAQLLLTTLIS     68
BobLFG4      ..........MHKWN..LPYRKDDVESGGNSGERPLYPTMLESP.ELRWGFIRKVYSIIAFQLLLTIAVA     57
BobLFG3      ..........MYQWN..LPYRKDDLEAGGGSRSRPLYPTMHETP.ELRWGFIRKVYSIIAFQLLATIAVS     57
BobLFG1      MENHSDWIIHCWIFS..TTMEKSDIESGVVIGGKELYPKMTESP.ELRWAFTRKVYAILTLQLIVTVGVS     67
BobBI1.2     ..........MESFS.....SFFDSQPGSRSWSYESLKNLHQIS.PSVQNHLKRVYLTLCCALVASAFGA     54
BobBI1.1     ..........MDSFS.....SFFDSQPGSRSWSYDSLKNLRQIS.PSVQNHLKRVYLTLCCALVASAFGA     54

BobBIL4      ATVVKVHPISVFFTTTSAGFALYILVIFTPLIVMCPLYYYHQKHPVNYLLLAIFTVSLAFAVGLTCAFTS    118
BobLFG5.3    AVVVLNPPVNDLLTGSPG...LLLFLCIIPFVLIWPLHVYHQKHPVNLILLALFTISLSFTVGVSCAMTE    135
BobLFG5.1    AVVVLNPPVNDVLTGSPG...LLLFLCIIPFVLIWPLHVYHQKHPVNLILLSLFTISLSFTVGVSCAMTE    135
BobLFG5.2    AVVVLNPPVNDVLTGSPG...LLLFLCIIPFVLIWPLHVYHQKHPVNLILLSLFTISLSFTVGVSCAMTE    135
BobLFG4      ATVVTVRPIAVFFSTTSAGLALWIVLIITPLLVLCPLYYYHQKHPVNYLFLGIFTVALAFAVGLSCAFTS    127
BobLFG3      ATVVTVRPIALFFATTGAGLGLYIVIIITPFIVLCPLYYYHQKHPVNYLLLGVFTLALAFVVGLTCAFTN    127
BobLFG1      SVVFFVGEISVFITTTTSGLVVFFVSLLLPLLMLWPLIVFAKKHPVNLIILMLFTLSISFAVGLCCSFSK    137
BobBI1.2     YLHVLWNIGGILTTIACCG........TMIWLLSCPPYEQQKRLSLLFLSAVLEGASVGPLIKVAVDFDP    116
BobBI1.1     YLHVLWNIGGILTTIGCFG........SMIWLLSCPPYEQQKRLSLLFLSAVLEGASVGPLIKVAVDFDP    116

BobBIL4      GKVILEAVILTAVVVVSLTLYTFWAAKRGHDFNFLGPFLFGAVIVLMVFSFIQILFPLGKISVMIYGCLA    188
BobLFG5.3    GRIVLEALILTLSVVGSLTAYTFWAAKKGKDFSFLGPILFTSLIILVVTSFMQMFFPLGPTSVAIYGGVS    205
BobLFG5.1    GRIVLEALILTLSVVGSLTAYTFWAAKKGKDFSFLGPILFTSLIILVVTSFMQMFFPLGPTSVAIYGGIS    205
BobLFG5.2    GRIVLEALILTLSVVGSLTAYTFWAAKKGKDFSFLGPILFTSLIILVVTSFMQMFFPLGPTSVAIYGGIS    205
BobLFG4      GKVILESAILTTVVVLSLTFYTFWAAKKGYDFNFLGPFLFGAIIVLMVFALIQIFFPLGRISVMIYGCLA    197
BobLFG3      GKVILESAILTTVVVLSLTVYTFWAAKRGYDFNFLGPFLFGALIVLVVFAMIQVFFPLGRTSVMIYGFLA    197
BobLFG1      GKIVLEAAVLTATMVVGLTIYTFWAVRRGHDFSFLAPFLFGSLLIILVFATIQVFHPLGKLSSMIFSCVV    207
BobBI1.2     S.ILITAFVGTAIAFICFSGAAMLARRR..EYLYLGGLLSSGLSMLMWLQFASSIFGGSASIFKFELYFG    183
BobBI1.1     S.ILITAFVGTAIAFICFSGAAMLARRR..EYLYLGGLLSSGLSMLMWLQFASSIFGGSASIFKFELYFG    183

BobBIL4      SIIFCGYIVYDTDNLIKRHSY..DEYIWAAISLYLDVINLFLSLL..TLLRAADS...............    239
BobLFG5.3    ALVFCGYIVYDTDNLIKRFTY..DEYILASVALYLDILNLFLTIL..RILRQGDN...............    256
BobLFG5.1    ALVFCGYIVYDTDNLIKRFTY..DEYILASVALYLDILNLFLTIL..RILRQGDN...............    256
BobLFG5.2    ALVFCGYIVYDTDNLIKRFTY..DEYILASVALYLDILNLFLTIL..RILRQGDN...............    256
BobLFG4      SIIFCGYIVYDTDNLIKRYSY..DEYIWAAVSLYLDIINLFLSLL..TIFRAAES...............    248
BobLFG3      SVIFCGYIVYDTDNLIKRYTY..DEYIWAAVSLYLDIINLFLSLL..TIFRALQR...............    248
BobLFG1      SVCFCGYIIYDTNQLIKKLNY..DEYIHAAISLYLDVINLFLNLVGENMIHAYVQCGDLSTESLKLCLVS    275
BobBI1.2     LLIFVGYMVVDTQEIIEKAHLGDMDYVKHALTLFTDFVAVFVRVLIIMLKNSADKEEKKKKRRN......    247
BobBI1.1     LLIFVGYMVVDTQDIIEKAHLGDMDYVKHSLTLFTDFVAVFVRVLIIMLKNSADKEDKKKRRRN......    247

BobBIL4      .                                                                         239
BobLFG5.3    .                                                                         256
BobLFG5.1    .                                                                         256
BobLFG5.2    .                                                                         256
BobLFG4      .                                                                         248
BobLFG3      .                                                                         248
BobLFG1      V                                                                         276
BobBI1.2     .                                                                         247
BobBI1.1     .                                                                         247
```

Figure S1. Multiple sequence alignment of nine Bax inhibitor-1 proteins in *Brassica oleracea var. botrytis*.

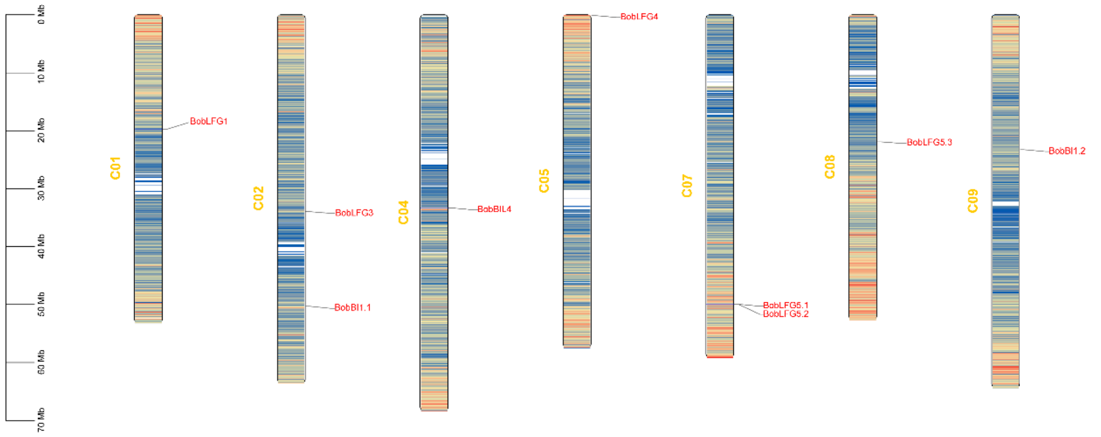

Figure S2. Schematic representation of the chromosome distribution of *BI-1*. The measurements on the left indicate the length of the chromosomes; the chromosome numbers are to the left of each chromosome; and the vertical bar is the cauliflower chromosome.

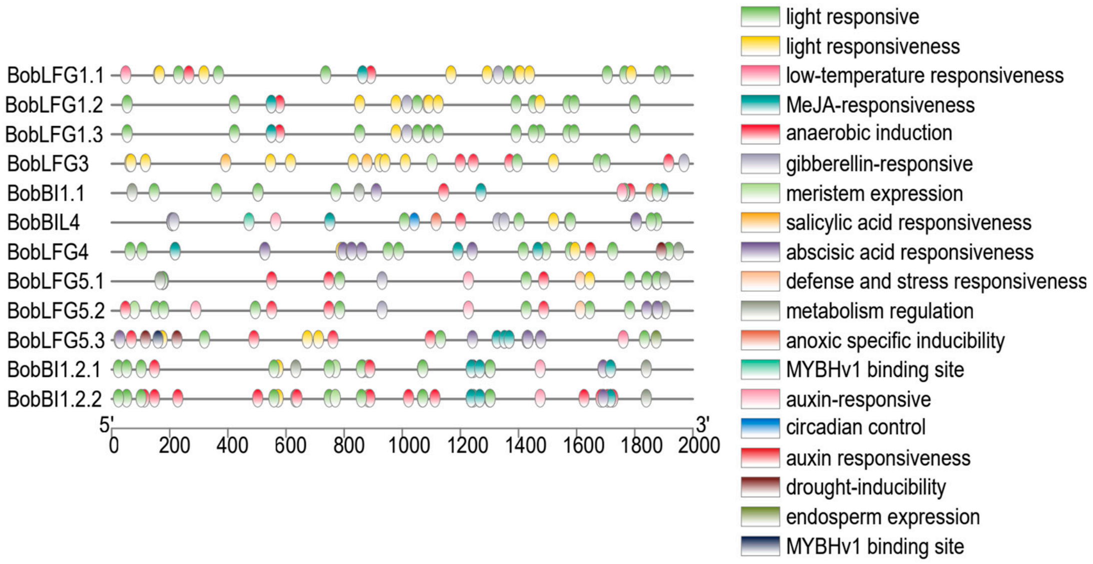

Figure S3. The distributions of predicted cis-acting elements in cauliflower *BI-1* genes, with different colors representing cis-acting elements with distinct functions.

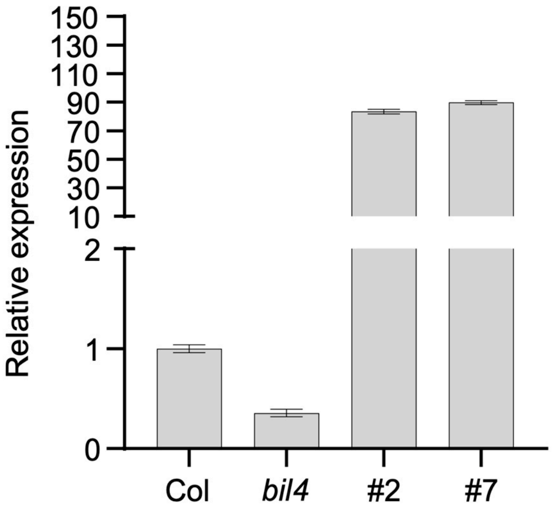

Figure S4. Relative expression levels of the *BIL4* gene in wild-type Arabidopsis (Col) and *BobBIL4* in *bil4* mutant lines. The results show that the expression levels of *BobBIL4* in the transgenic lines #2 and #7 are approximately 84 and 90 times higher, respectively, than the *BIL4* expression in the wild type. The *bil4* mutant line shows significantly reduced expression compared to the wild type. Data are presented as the mean ± standard error (SE) from three independent experiments.

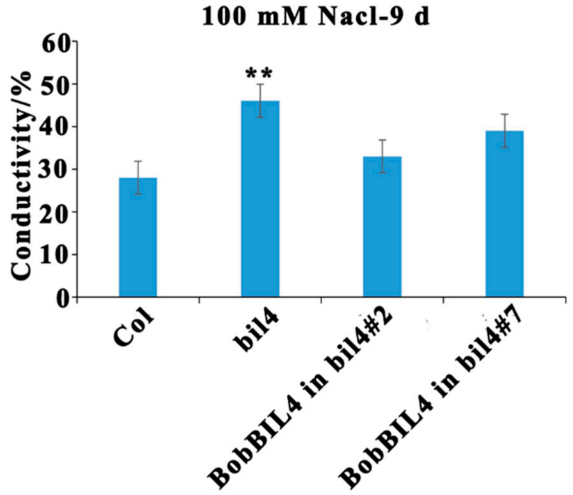

Figure S5. *BobBIL4* reduced cell death induced by salt stress. The data are presented as mean ± SD. Significant differences compared to *bil4* plants are indicated by asterisks (*$p < 0.05$, n = 60).

Table S1. RT-PCR primers for quantitative expression analysis.

primer name	sequence (5'to3')
BobACTIN-F	CGGGATTGGTGATAGGATGAG
BobACTIN-R	GCTGGAAAGTACTGAGGGAAG
BobBI1.1-F	GGCAGTTGATTTTGACCCAAG
BobBI1.1-R	GACAAGCCAGATGAAAGCAG
BobBIL4-F	ATGACTTCAACTTCCTCGGTC
BobBIL4-R	GGGAAGAGAATCTGGATGAAGG
BobLFG5.3-F	TCTAACCGCATACACTTTCTGG
BobLFG5.3-R	ATGAGGCTGGTGAAGAGAATG
BobBI1.2-F	GCTCTCACTTCTCTTCCTCTC
BobBI1.2-R	CTTGGGTCAAAATCAACAGCC
